# Cell-Free Systems: A Proving Ground for Rational Biodesign

**DOI:** 10.3389/fbioe.2020.00788

**Published:** 2020-07-24

**Authors:** Nadanai Laohakunakorn

**Affiliations:** School of Biological Sciences, Institute of Quantitative Biology, Biochemistry, and Biotechnology, University of Edinburgh, Edinburgh, United Kingdom

**Keywords:** cell-free synthetic biology, cell-free protein synthesis, *in vitro* transcription translation, model-guided design, rational design

## Abstract

Cell-free gene expression systems present an alternative approach to synthetic biology, where biological gene expression is harnessed inside non-living, *in vitro* biochemical reactions. Taking advantage of a plethora of recent experimental innovations, they easily overcome certain challenges for computer-aided biological design. For instance, their open nature renders all their components directly accessible, greatly facilitating model construction and validation. At the same time, these systems present their own unique difficulties, such as limited reaction lifetimes and lack of homeostasis. In this Perspective, I propose that cell-free systems are an ideal proving ground to test rational biodesign strategies, as demonstrated by a small but growing number of examples of model-guided, forward engineered cell-free biosystems. It is likely that advances gained from this approach will contribute to our efforts to more reliably and systematically engineer both cell-free as well as living cellular systems for useful applications.

## 1. Introduction

A basic aim of synthetic biology is to design and construct biological systems which perform a given function. An extension of this, inspired by common engineering practice, is to additionally demand that the systems perform robustly, predictably, and with quantitative precision. Some practitioners of synthetic biology explicitly adopt the conventional engineering approach of rational design, where a system is constructed predictively (Endy, 2005; Heinemann and Panke, 2006). In contrast to non-biological engineering, synthetic biological systems are also open to the possibility of evolutionary design (Arnold, 1998), where function is obtained through directed evolutionary screens. It is still an open question as to whether or not a purely rational engineering approach can ultimately be successfully applied to engineer complex biomolecular systems (Davies, 2019).

A fully rational approach adopts all conventional engineering principles, such as standardization and quantitative characterization of parts, mathematical models to describe their behavior, and abstraction which allows hierarchical assembly of parts into modules, subsystems, and systems (Endy, 2005; Arkin, 2008; Canton et al., 2008). For any system of non-trivial complexity, this approach relies on computational methods to enable predictive design (MacDonald et al., 2011).

To a large extent, strict adherence to this approach has not yet been widely successful in synthetic biology, with a few notable exceptions (e.g., Nielsen et al., 2016). Typically, biodesign involves multiple iterations through a so-called “design-build-test-learn” (DBTL) cycle. While eventually a functioning system is produced, the path to get there is not directly through predictive design, but rather informed trial-and-error. The current necessity of DBTL cycles is due to partly to the fact that the complexity associated with biomolecular systems eludes simplifying black-box approximations common in other physical scenarios. Additionally, interrogating biosystems with controlled inputs and perturbations is difficult, and system parameters can be context-dependent and varying. However, with the emergence of high-throughput automation and biofoundries (Hillson et al., 2019), the promise is that DBTL efforts will ultimately enable fully predictive, rational biodesign.

**Figure 1:**
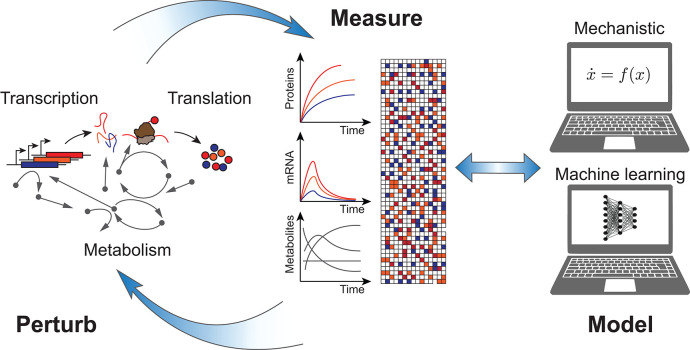
Rational computer-aided biodesign with cell-free systems: cell-free gene expression systems perform *in vitro* transcription, translation, and metabolism in reactions which are open and transparent to measurement and perturbation. Data sets generated are kinetically resolved and high-throughput, facilitating development of both mechanistic as well as machine learning models.

Cell-free systems (Garenne and Noireaux, 2019) can contribute in several ways to improve the design process of synthetic biological systems, which span scales from the molecular (genetic regulatory elements, proteins, enzymes), to the systemic (gene regulatory and metabolic networks), and all the way to the extracellular levels (synthetic cells, communication, self-assembly). First, they can accelerate DBTL cycles through rapid prototyping (Chappell et al., 2013; Niederholtmeyer et al., 2015; Takahashi et al., 2015). Second, they can be used efficiently for *in vitro* directed evolution (Contreras-Llano and Tan, 2018). In this Perspective I would like to focus on a third contribution, and suggest that they offer an ideal proving ground to test the approach of rational computer-aided biodesign as applied to biomolecular systems (Figure 1). In particular, they present features which overcome some of the difficulties associated with engineering living cells, and so can be used to more easily develop and calibrate mechanistic models, as well as generate sufficient data for machine learning approaches.

To understand their strengths and weaknesses in the context of synthetic biology, it is first important to consider the differences between cell-free and living cellular systems.

## 2. Biophysical Differences Between Cell-Free and Cellular Systems

Today, cell-free technology generally refers to cell-free protein synthesis (CFPS), which rests on the foundational processes of *in vitro* transcription and translation (Silverman et al., 2019a; Laohakunakorn et al., 2020). Strictly speaking, CFPS belongs to the much broader field of *in vitro* reconstitution, which consists of recapitulating biological processes outside of the living cell. This involves combining relevant enzymes (either purified or extracted in crude cellular lysate) with a reaction mixture containing substrates, cofactors, and specific ionic and pH conditions. Constructing such a reaction isolates specific biological processes, and has historically served as a key approach to elucidate molecular biological mechanisms, including deciphering the genetic code itself (Nirenberg and Matthaei, 1961; Zubay, 1973). While this article will focus predominantly on bacterial cell-free systems due to their current widespread use, cell-free systems have also been successfully prepared from a number of prokaryotic and eukaryotic organisms (Perez et al., 2016).

In addition to developing fundamental understanding, this approach also enables technological applications: examples of these are the use of CFPS to carry out *in vitro* biomanufacturing, where the production of exogenous protein is advantageously decoupled from cellular growth (Karim and Jewett, 2018; Gregorio et al., 2019); and biosensing, where robust, lyophilized cell-free gene circuits can be activated and used to detect environmental contaminants and pathogens directly in the field (Pardee et al., 2014).

One predominant viewpoint of cell-free systems is that they are cellular mimics. The crude cellular lysate is a representation of the cellular cytosol, and contains, in addition to transcription and translation, a number of intact and functional core metabolic pathways (Kim and Swartz, 2001; Kim and Kim, 2009). Thus, cell-free systems have been successfully used as a prototyping platform for synthetic biology, a so-called “cellular breadboard” where synthetic gene circuit designs can be quickly implemented, validated, and ported back into a living cell (Siegal-Gaskins et al., 2014; Garamella et al., 2016). The success of this approach relies on a basic similarity between the cell-free and cellular environments, an assumption that has been verified in a number of notable examples (Chappell et al., 2013; Niederholtmeyer et al., 2015; Borkowski et al., 2018; Halleran and Murray, 2018; Hu et al., 2018).

On the other hand, cell-free systems do contain fundamental differences from cells. In addition to being non-living, there are a number of key biophysical differences. Below I highlight these, and consider their consequences in the context of the implementation of a generic synthetic gene regulatory network (GRN). In some cases, strategies for making the system more “lifelike” by bottom-up construction are briefly discussed.

1. **Accessible system:** Without a barrier between the reaction and the environment, the cell-free reaction is transparent to observation and perturbation, allowing the reaction conditions to be adjusted at will. This property can be leveraged, for example, to change redox environments to promote disulphide bond formation (Matsuda et al., 2013). The kinetic progress of reactions can be followed using fluorescence from proteins and mRNA, as well as real-time metabolomic profiling, which has allowed the internal metabolism of cell-free systems to be dissected at high resolution (Bujara et al., 2011; Vilkhovoy et al., 2019). For GRN design, parameters, such as dissociation and kinetic constants between a transcription factor and promoter may be measured *in situ* (Geertz et al., 2012; Swank et al., 2019), and perturbations applied to the reaction composition to facilitate parameter identification and model selection (Hu et al., 2015; Moore et al., 2018). These key properties of controllable inputs, perturbations, and consistency between conditions where parameter measurement and system operation take place directly address challenges faced in engineering living cells. Crucially, this enables a close coupling of cell-free experiments and computational models.
2. **Dilute, well-mixed reaction environment:** The lack of compartmentalization is also related to a number of other physical effects, including a loss of stochasticity, slower enzymatic rates, a reduced level of macromolecular crowding, a loss of spatial organization, and a loss of membrane-associated processes [although lysates can contain inverted membrane vesicles which permit oxidative phosphorylation (Jewett et al., 2008)]. A useful consequence of such a simplified reaction environment is that the system can be described with deterministic kinetics; a practical side-effect is that exogenous protein aggregation is minimized, which facilitates bioproduction. In order to recreate more lifelike reaction environments, there is much ongoing effort to encapsulate cell-free reactions in a variety of compartments including liposomes, polymersomes, and droplets, as well as introducing crowding and organization into cell-free systems (Laohakunakorn et al., 2020).
3. **Relaxation to equilibrium:** Living cells are maintained in a homeostatic, non-equilibrium steady state by a constant flux of energy and metabolites through the system, while cell-free reactions relax to biochemical equilibrium as the reaction proceeds. This sets a limit on the lifetime of the cell-free reaction. The lifetime may be extended by engineering a more homeostatic metabolic system [for instance, rationally-designed *in vitro* metabolic systems can operate autonomously for days (Korman et al., 2017)], but ultimately to maintain cell-free systems in a steady state, an energy and metabolite flux must be set up between the system and environment. This can be achieved using continuous flow or continuous exchange reactors (Spirin et al., 1988; Niederholtmeyer et al., 2013; Karzbrun et al., 2014), or by compartmentalizing and coupling the reaction to transport processes (Noireaux and Libchaber, 2004). A consequence of limited lifetime is that after a few hours, any synthetic cell-free gene circuit ceases to be functional. Thus, recent efforts have focused on extending reaction lifetimes (Caschera and Noireaux, 2015) as well as accelerating the computational and output steps in the circuit (Alam et al., 2019).
4. **No regulation:** While living cells are actively regulated at multiple levels of organization, from molecular- to network-scale, cell-free systems contain no active regulatory mechanisms. This simplifies the identification and measurement of host-chassis interactions, allowing resource allocation within the cell-free system to be elucidated in detail (Siegal-Gaskins et al., 2014; Gyorgy and Murray, 2016; Borkowski et al., 2018; Halter et al., 2018). On the other hand, cell-free systems lose the robustness conferred by homeostasis (Lewis et al., 2014). They are thus sensitive to effects which would otherwise be regulated, for example partial degradation products (Kim and Winfree, 2011), stochasticity in gene expression (Karig et al., 2013), and variable partitioning of reactants during system encapsulation (Altamura et al., 2018). This property may thus be an impediment to predictive design.
5. **No self-regeneration:** Self-regeneration is a defining hallmark of life (Luisi et al., 2006), and cell-free systems do not regenerate their components, implying that the lifetime of cell-free reactions is also limited by enzyme stability (Stögbauer et al., 2012), in addition to resource depletion and metabolic arrest. The possibility of programming regeneration directly in the cell-free system leads to the tantalizing prospect of a cell-free system capable of maintaining its components, which could form the basis of an engine to power artificial cells (Schwille et al., 2018).
6. **No replication:** In addition to not regenerating their components, cell-free systems also do not replicate their genetic material. This has been considered an opportunity for bottom-up reconstruction, from early demonstrations of *in vitro* replication of plasmids and viral DNA in prokaryotic and eukaryotic lysates (Diaz and Staudenbauer, 1982; Li and Kelly, 1984; Stillman and Gluzman, 1985) to more recent studies involving phi29 DNA polymerase (Sakatani et al., 2015; van Nies et al., 2018), which have culminated in the replication of up to 116 kb of DNA in the PURE system (Libicher et al., 2020). Lack of replication implies genetic stability of introduced DNA, unlike living cells which can mutate away exogenous gene circuit function. Steady-state *in vitro* replication of nucleic acids would form a necessary subsystem of self-replicating artificial cells as well as enable *in vitro* evolutionary studies (Meyer et al., 2012).

These properties have influenced the approaches used in cell-free engineering. In particular, the accessibility of the reaction environment has made cell-free systems particularly suited for rational biodesign strategies, as will be discussed next.

## 3. Rational Biodesign Strategies for Cell-Free Synthetic Biology

### 3.1. Model-Guided Design

The most ambitious approach to rational biodesign uses a quantitative and predictive model to guide the design process, adopting workflows from well-established fields, such as electrical and aerospace engineering. For synthetic biology, the largest obstacles to this involve unknown, uncharacterized, or changing interactions among biomolecular components, and the difficulty of accessing and perturbing system components. In general, we can envisage two broad approaches which aim to mitigate this knowledge gap in cell-free systems: a “bottom-up” approach, where purified, reconstituted systems are constructed one component at a time, allowing interactions to be taken into account as they arise; and a “top-down” approach, where crude cellular lysates are interrogated and potentially modified to remove unwanted interactions, exposing the minimal system beneath. These approaches mirror the bottom-up and top-down approaches to the construction of artificial cells, with the final result being a minimal system that is maximally understood.

Recently, efforts have been made to combine the development of reconstituted cell-free systems with mathematical modeling (Mavelli et al., 2015; Matsuura et al., 2017, 2018; Carrara et al., 2018; Doerr et al., 2019). Reconstituted systems are composed of purified cellular enzymes and an energy solution, mixed together in a known composition, and are available commercially [e.g., commonly-used variants based on the PURE system (Shimizu et al., 2001)]. Compared to lysates, reconstituted systems are dramatically simplified. In principle, since the exact system composition is known, a model incorporating all predicted interactions can be written down. In practice, it is infeasible to calibrate such fine-grained models to experimental measurements, although properties, such as robustness of the system can be investigated *in silico* (Matsuura et al., 2017). Current coarse-grained models are generally not sufficient to globally capture all observed experimental effects (Doerr et al., 2019). The overarching aim is therefore to search for computational models of appropriate granularity which can describe all experimental observations, and yet remain feasible for calibration. The success of this is likely to be borne out through approaches which combine automation and high-throughput measurements with improved cost-efficient methods for preparing recombinant systems (Lavickova and Maerkl, 2019).

The top-down, systems-level approach aims to develop mechanistic understanding by interrogating lysates, which contain significantly more unknowns. The ‘black-box’ of lysates has slowly been opened over the last two decades, motivated by a desire to improve productivity and lifetime of the system (Silverman et al., 2019b). Using a combination of strain engineering and data from biochemical and metabolic analyses, it is now possible to rationally redirect metabolic flux and energy usage. Energy regeneration schemes of increasing complexity have been developed in order to improve lysate reaction lifetime and yield, proceeding initially from single-step (Zubay, 1973; Kigawa et al., 1999) to multi-step pathways which regenerate ATP using enzymes present within the extract (Kim and Swartz, 1999, 2001; Jewett and Swartz, 2004; Sitaraman et al., 2004; Calhoun and Swartz, 2005; Jewett et al., 2008; Caschera and Noireaux, 2015). While the complexity of lysates is considerable, in contrast to cellular systems biology, cell-free systems are amenable to essentially unconstrained perturbation, which greatly facilitates model testing and validation. This has been demonstrated by a number of modeling studies of increasing sophistication (Karzbrun et al., 2011; Stögbauer et al., 2012; Tuza et al., 2015; Gyorgy and Murray, 2016; Nieß et al., 2017; Marshall and Noireaux, 2019), as well as notable examples of model-guided forward engineering of genetic circuits (Hu et al., 2015, 2018; Agrawal et al., 2019; Lehr et al., 2019; Westbrook et al., 2019). Recent development of integrated gene expression and metabolic models have elucidated the factors limiting CFPS (Wayman et al., 2015; Vilkhovoy et al., 2018, 2019; Horvath et al., 2020), suggesting that combined computational and experimental metabolomic studies are poised to contribute significantly to our understanding of CFPS in lysates.

### 3.2. Control Theoretic Approaches for Robust Operation

Even if all interactions could be measured, many are unlikely to remain constant with time. Additionally, cell-free reactions operate dynamically, in an equally dynamic, fluctuating environment. It is clear, then, that knowledge of interactions is not generally sufficient to ensure robust performance.

Control engineering attempts to maintain the performance of a dynamic system within certain specified bounds, while parts of the system are subject to uncontrolled disturbances. A specific example of this is reference tracking using feedback control, where an output, such as the gene expression level follows a reference signal despite the presence of perturbations. Achieving this requires the system to sense the reference as well as its output, which involves redirecting the output back into the system in a feedback loop. Another example is buffering outputs against upstream variability using feed-forward regulators which balance each other's activity. Feedback and feedforward loops are ubiquitous in natural biological systems, making it a natural extension to develop synthetic biology within a control theoretic framework (Vecchio et al., 2016; Del Vecchio et al., 2018; Hsiao et al., 2018; Baetica et al., 2019).

Feedback control has been successfully implemented in a number of *in vivo* examples, for instance to regulate exogenous gene expression in response to burden (Ceroni et al., 2018), or to control growth rate using robust perfect adaptation (Aoki et al., 2019). Feedforward architectures have also been used to control variations in the amount of DNA template present in cells (Bleris et al., 2011). In the context of control circuits, a significant advantage of cell-free over cellular systems is that they are free from biological noise, and operate in the deterministic regime. Despite these benefits, surprisingly little work has been carried out on cell-free control; recent examples, including a computational study (Agrawal et al., 2018) and an experimental demonstration of a feedback integral controller based on molecular sequestration (Agrawal et al., 2019), as well as feedforward loop circuits (Guo and Murray, 2019), suggest that such approaches are starting to become more widespread within the cell-free community.

### 3.3. Active Learning

While a mechanistic or phenomenological model may lead to transparent understanding of the system, they are not the only models offering sufficient predictive capabilities for rational design. Statistical or non-parametric models, developed from data using machine learning approaches, can be equally or more strongly predictive, albeit with the well-known challenges of interpretability (Doshi-Velez and Kim, 2017).

An example problem is to determine the composition of a cell-free reaction to maximize its protein productivity. In the absence of a predictive model fully connecting all its components to protein output, Caschera et al. (2018) used an evolutionary design of experiments approach to iteratively train an ensemble neural network model to optimize conditions for cell-free protein synthesis. More recently Borkowski et al. (2020) trained a similar model on ~4,000 reactions, improving yields by 34 times. Importantly, they discovered a training dataset of only 20 compositions which was informative enough to allow the model to generalize its predictions to different lysates and conditions. These examples demonstrate that the throughput afforded by cell-free systems is sufficient for informing data-driven modeling approaches.

Data-driven cell-free techniques could also potentially be applied to other long-standing questions in systems biology, for instance, determining the mapping of sequence to phenotype for a genetic element (Cuperus et al., 2017; Sample et al., 2019). Cell-free implementations of massively parallel reporter assays, perhaps using droplet microfluidic technology to maintain genotype-phenotype linkage, may yield datasets of sufficient quality and size to contribute to this problem.

## 4. Conclusions

Cell-free systems are ideally suited for rational engineering approaches: their open reactions facilitate construction and validation of mechanistic and phenomenological models, and their throughput allows them to generate sufficient data to train machine learning models. Developing these models, and designing experiments to calibrate and validate them, are general strategies which can be tested on the cell-free platform, but eventually also applied to the more challenging problem of engineering living systems. In this sense, cell-free systems can be thought of as a proving ground for rational design strategies.

Cell-free systems do however present unique challenges for predictive design. As discussed above, a lack of homeostasis can imply ultrasensitivity of cell-free reactions to various effects. It is also well-known that strong batch-to-batch variation of lysates can limit the predictability of results to within-batch repeats, constraining the usefulness of the approach; however recent efforts have been made to identify and control these effects (Cole et al., 2019; Silverman et al., 2019b). And finally, while examples were given of successful transfer of cell-free designs back into cellular hosts, the generality of this approach has so far remained unclear. These are all avenues for future research within the field.

In this Perspective, I have deliberately left out a discussion of directed evolution, a complementary and powerful strategy for biodesign. Cell-free systems have also been extensively deployed for *in vitro* evolution (Contreras-Llano and Tan, 2018), maintaining genotype-phenotype coupling through the use of display technologies and compartmentalization.

Reliable engineering of synthetic biological systems remains a great challenge, and it is likely that a number of complementary efforts including rational as well as evolutionary design, and cellular and cell-free systems, will be required to eventually achieve this grand goal.

## Author Contributions

NL conceived and wrote the article.

## Conflict of Interest

The author declares that the research was conducted in the absence of any commercial or financial relationships that could be construed as a potential conflict of interest.
